# Supplementary figures and images for: A conserved protein tyrosine phosphatase, PTPN-22, functions in diverse developmental processes in C. elegans
Source: PLoS Genet. 2024 Aug 22;20(8):e1011219. doi: 10.1371/journal.pgen.1011219 (PMC11373843; doi:10.1371/journal.pgen.1011219)

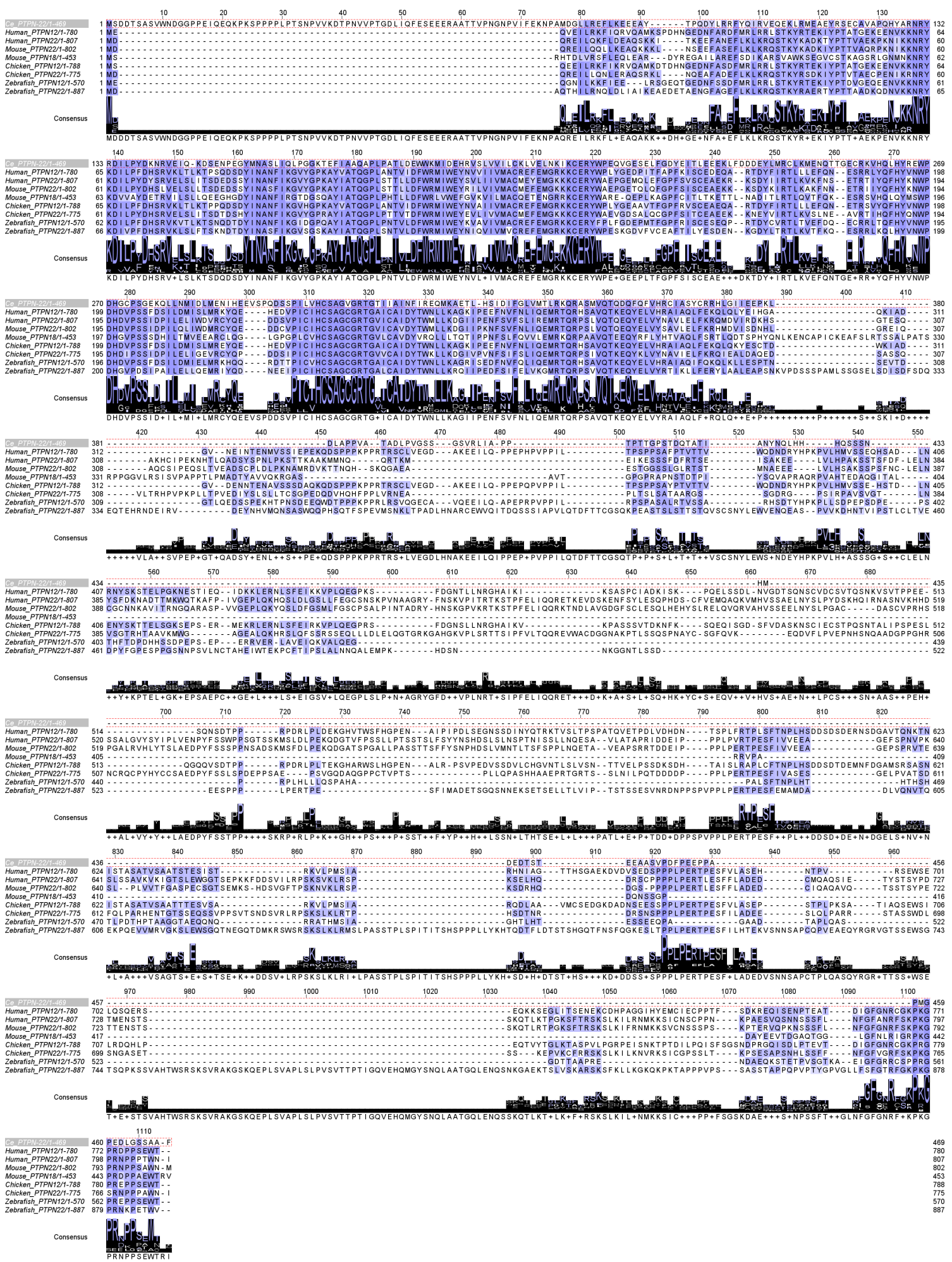

Supplement: S1 Fig — Jalview was used to visualize multi-sequence alignments among C. elegans PTPN-22, human PTPN12 and PTPN22, mouse PTPN22 and PTPN18, chicken PTPN12 and PTPN22, and zebrafish PTPN12 and PTPN22. Conserved residues, based on sequence homology, are highlighted in purple. A consensus sequence is provided below the sequence alignments. (PDF) [file pgen.1011219.s001.pdf]

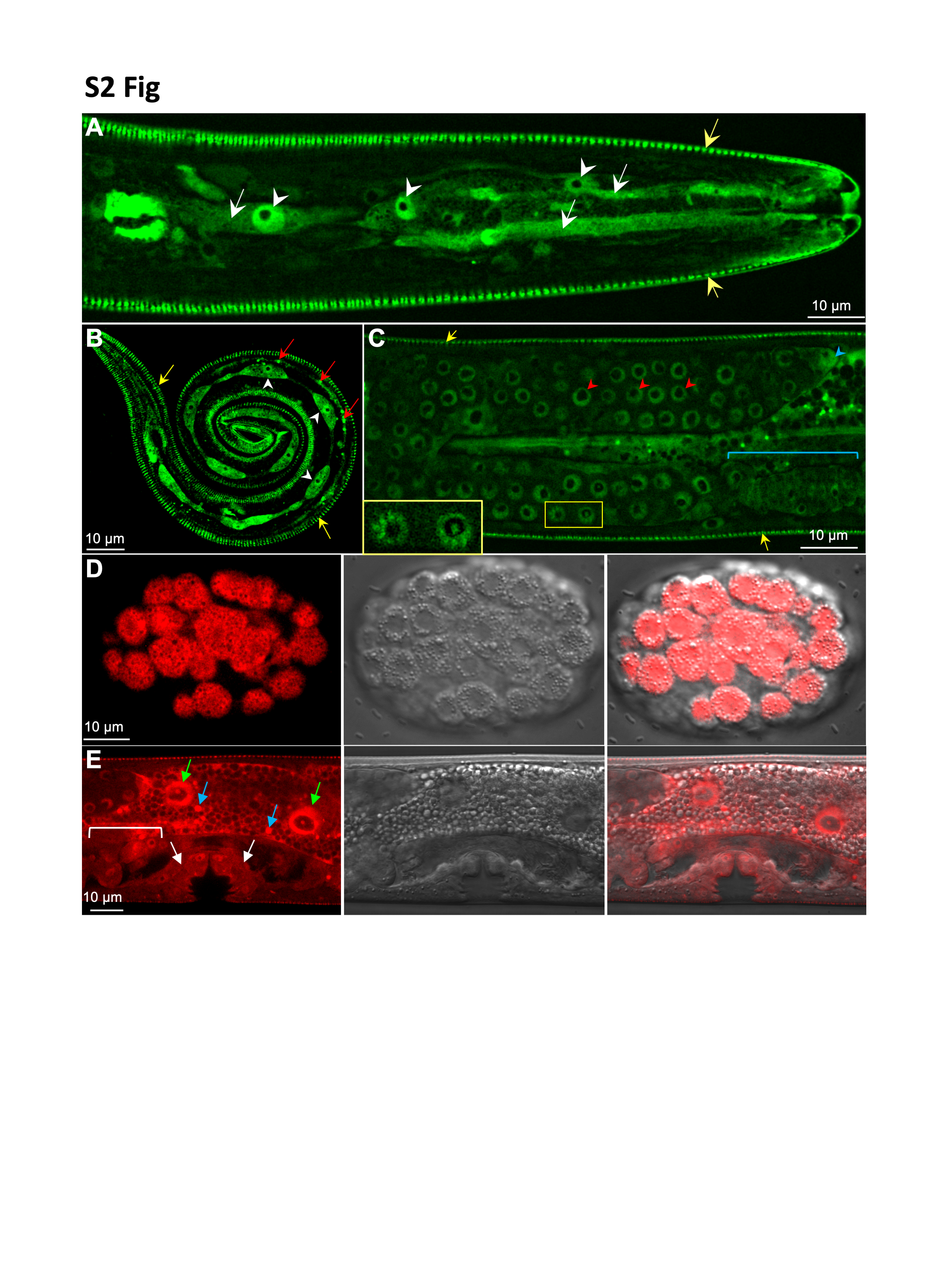

Supplement: S2 Fig — (A) A confocal microscopy image of the anterior body of a day-1 adult worm expressing PTPN-22::GFP. White arrows show examples of PTPN-22::EGFP in pharyngeal cells; white arrowheads show its expression in nuclear compartments of pharyngeal cells; yellow arrows indicate autofluorescence of the cuticle. (B) An L2 larva expressing PTPN-22::EGFP. White arrowheads indicate seams cells, which show expression in their cytoplasm and nuclei; red arrows indicate nerve cord cells; yellow arrows indicate autofluorescence of the cuticle. (C) Expression of PTPN-22::EGFP in the germline of an L4-stage worm including nuclear and perinuclear expression as indicated with red arrowheads; blue arrowhead indicates a distal tip cell; blue bracket indicates spermatheca; yellow arrows indicate autofluorescence of cuticle. Yellow box corresponds to the enlarged inset, which shows expression of PTPN-22::EGFP in the perinuclear region of germline nuclei. (D) Ubiquitous nuclear and cytoplasmic expression of PTPN-22::mScarlet in early embryonic cells (left), with the DIC (middle) and merged (right) images. (E) Nuclear and cytoplasmic expression of PTPN-22::mScarlet in vulval cells in an L4-stage worm (white arrows) with the DIC (middle) and merged (right) images. The green arrows show expression in intestinal nuclei; blue arrows show gut granule autofluorescence; white bracket indicates proximal somatic gonad cells. (PNG) [file pgen.1011219.s002.png]

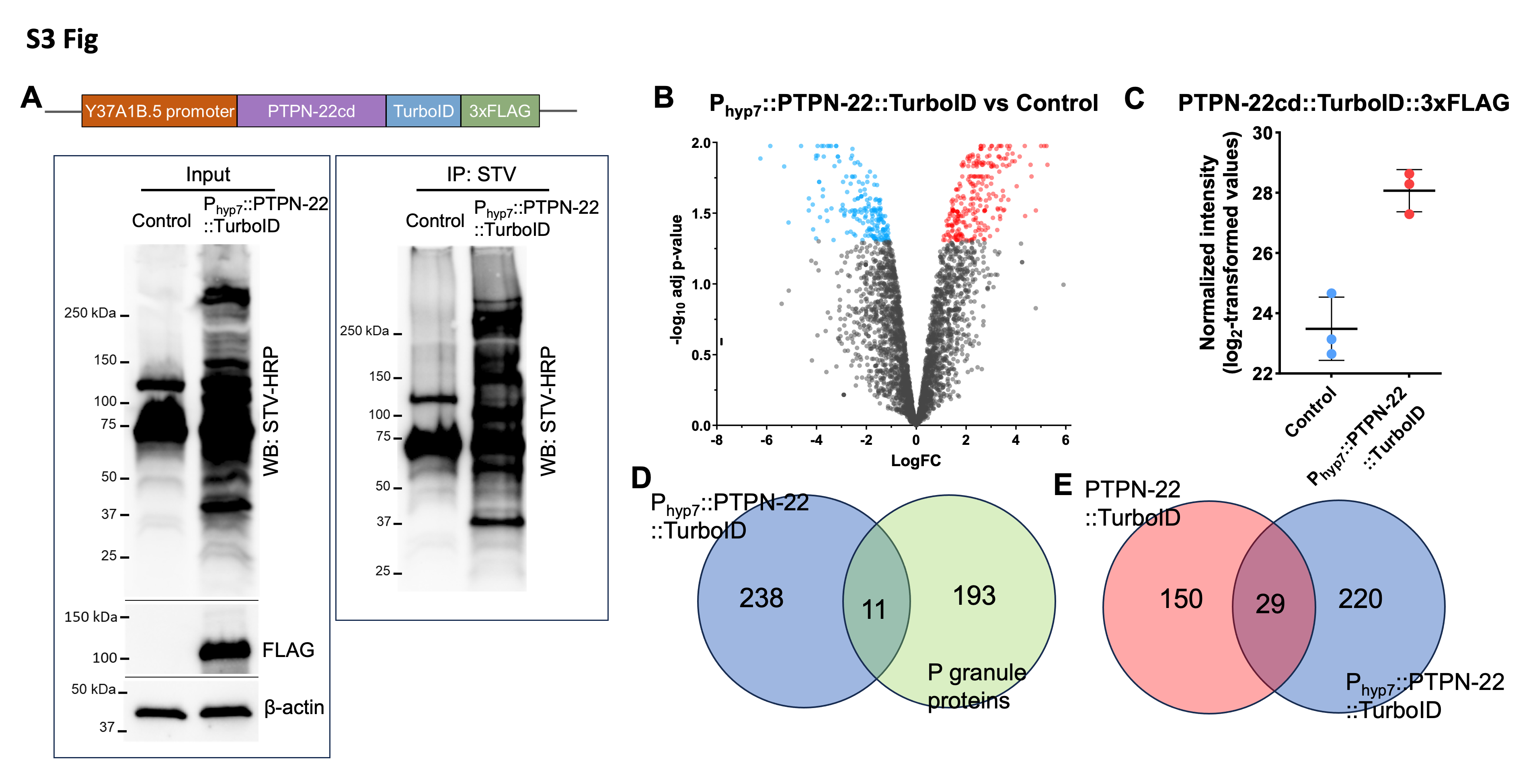

Supplement: S3 Fig — (A) Schematic showing relevant portion of the expression construct used to drive PTPN-22::TurboID in the major hyp7 epidermal syncytium. A ptpn-22 cDNA was fused to coding sequences for TurboID and a 3×FLAG tag (both placed at the C terminus) and expressed under the control of a hyp7-specific (Y37A1B.5) promoter. Western blot images of representative N2 and Phyp7::PTPN-22::TurboID samples show the biotinylated proteins in the input and pulldown (IP) fraction after blotting with streptavidin-HRP. The lower two blots on the left show Phyp7::PTPN-22::TurboID expression based on an antibody against FLAG (upper) and a loading control with an antibody against β-actin (lower). (B) The volcano plot shows the enrichment of proteins after LC-MS/MS analysis in the Phyp7::PTPN-22::TurboID samples (red) and in the N2 samples (blue). (C) The dot plot shows normalized intensity values of ectopically expressed PTPN-22cd::TurboID::3×FLAG versus the N2 control (three replicates each). Error bars represent standard deviation. (D, E) Venn diagram shows the overlap of enriched proteins between Phyp7::PTPN-22::TurboID samples and P granule proteins (D) and the overlap of enriched proteins between PTPN-22::TurboID samples and Phyp7::PTPN-22::TurboID samples (E) (see S4 File). cd, cDNA; STV, streptavidin. (PNG) [file pgen.1011219.s003.png]

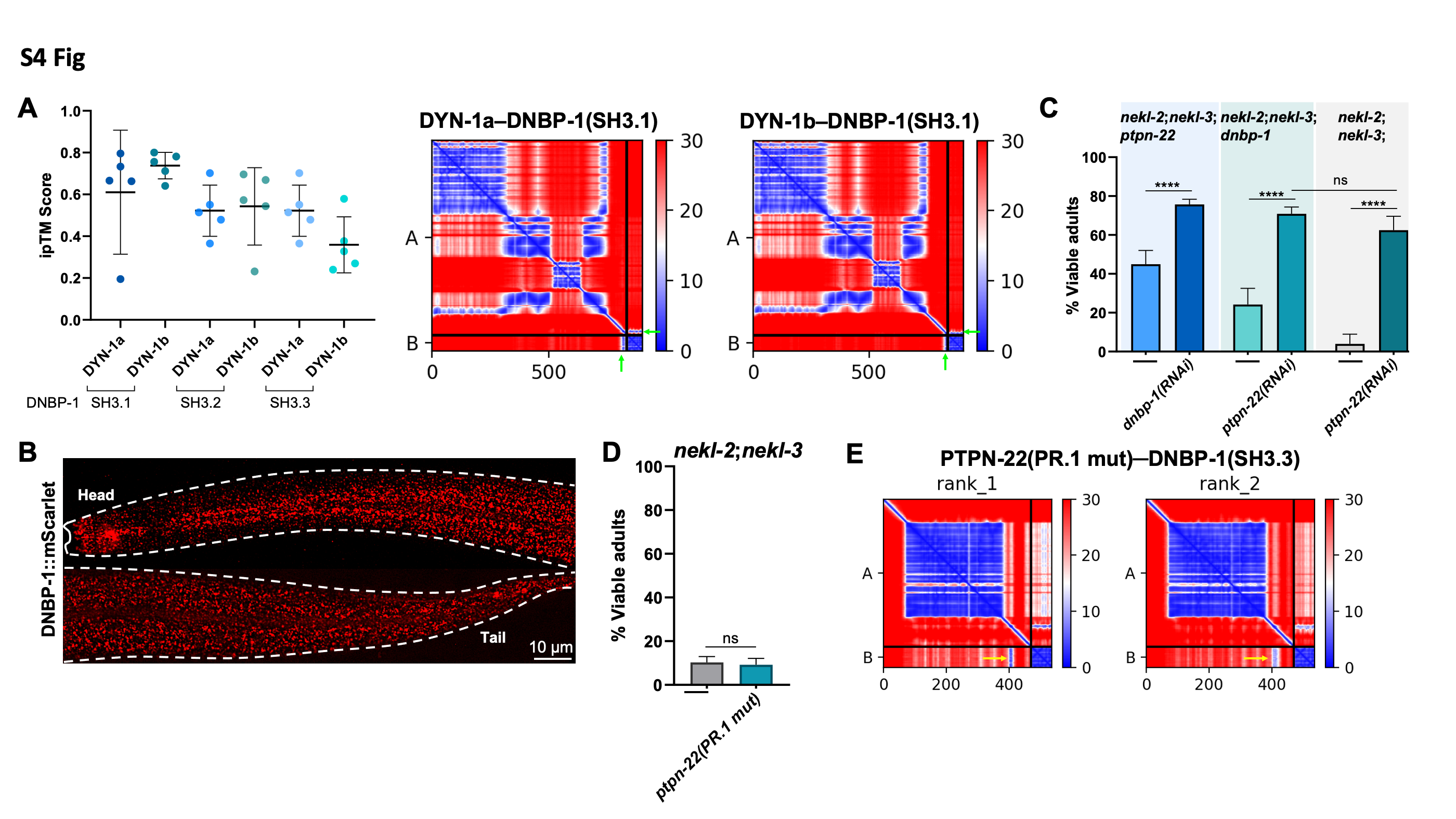

Supplement: S4 Fig — (A) ipTM scores for five different models generated using AlphaFold-multimer, each of which was used to determine predicted binding between the three SH3 domains (SH3.1, SH3.2, and SH3.3) of DNBP-1 and two isoforms of DYN-1 (DYN-1.a and DYN-1.b). Error bars represent standard deviation. PAE plots of the best models for DYN-1.a and DYN-1b interactions with DNBP-1(SH3.1) are shown with green arrows indicating the predicted proline-rich region of interaction at the C terminus of DYN-1.a and DYN-1.b. (B) Representative confocal images of day-1 adult worms expressing DNBP-1::mScarlet in the anterior and posterior side of the epidermis. (C,D) Bar graph showing the percentage of suppressed worms in the indicated backgrounds. Error bars represent 95% confidence intervals. Fisher’s exact test was used to calculate p-values; ****p < 0.0001; ns, not significant. (E) PAE plots showing the two highest-scoring Alphafold2 multimer interactions models (rank_1 and rank_2) of PTPN-22(PR.1mut) with the SH3.3 domain of DNBP-1. Yellow arrows indicate the predicted interacting region. Raw data are available in S7 File. (PNG) [file pgen.1011219.s004.png]

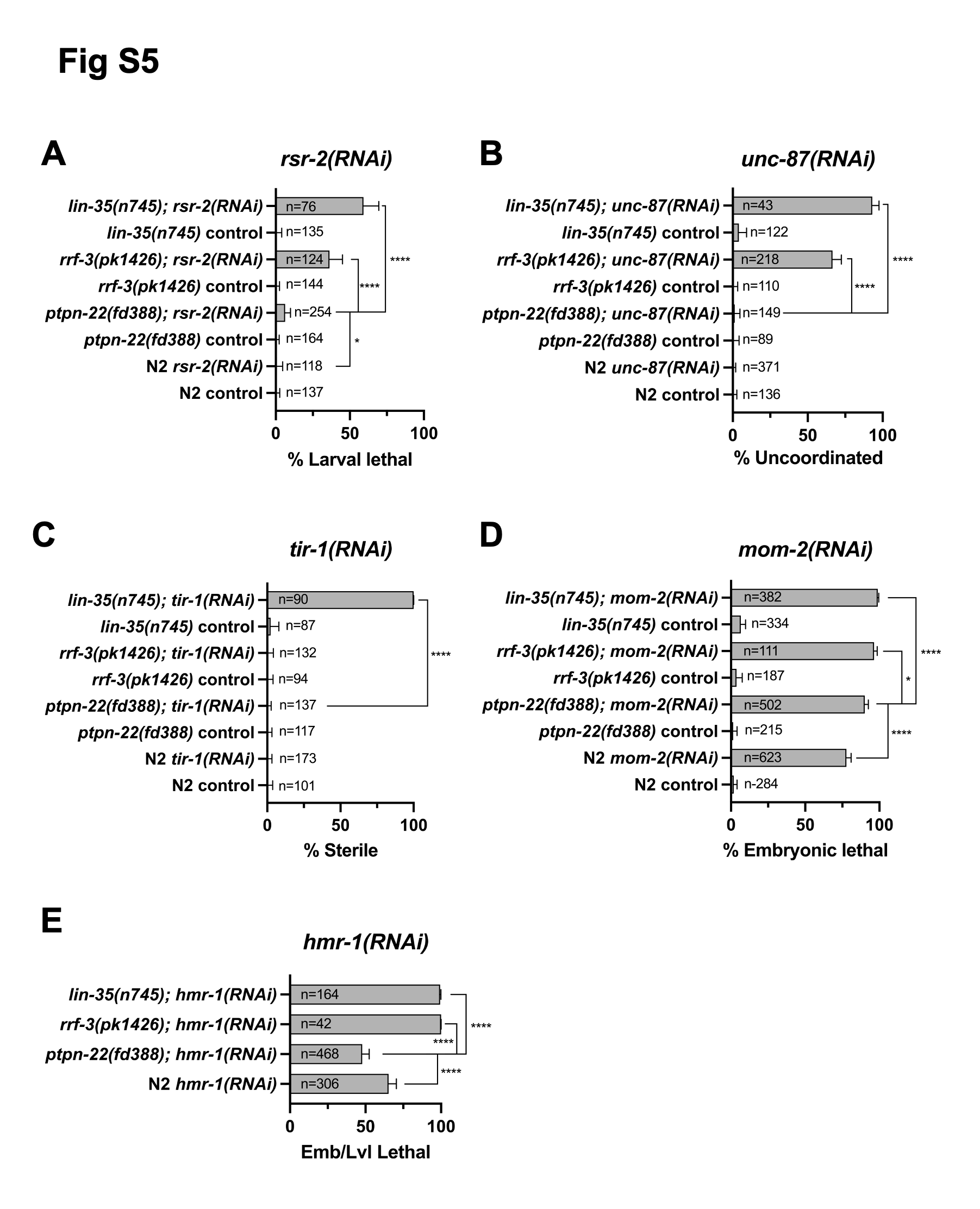

Supplement: S5 Fig — (A–E) P0 L4 larval of the indicated genotypes were placed on control GFP(RNAi) or experimental (rsr-2, unc-87, mom-2, tir-1, or hmr-1) RNAi-feeding plates for 24 h, and then moved to new RNAi-feeding plates and allowed to lay eggs for ~8 h before removing the P0 adults. F1 progeny, were scored ~1–4 days following the removal of P0 animals depending on the nature of the assessed phenotype. Raw data are available in the S7 File. (PNG) [file pgen.1011219.s005.png]

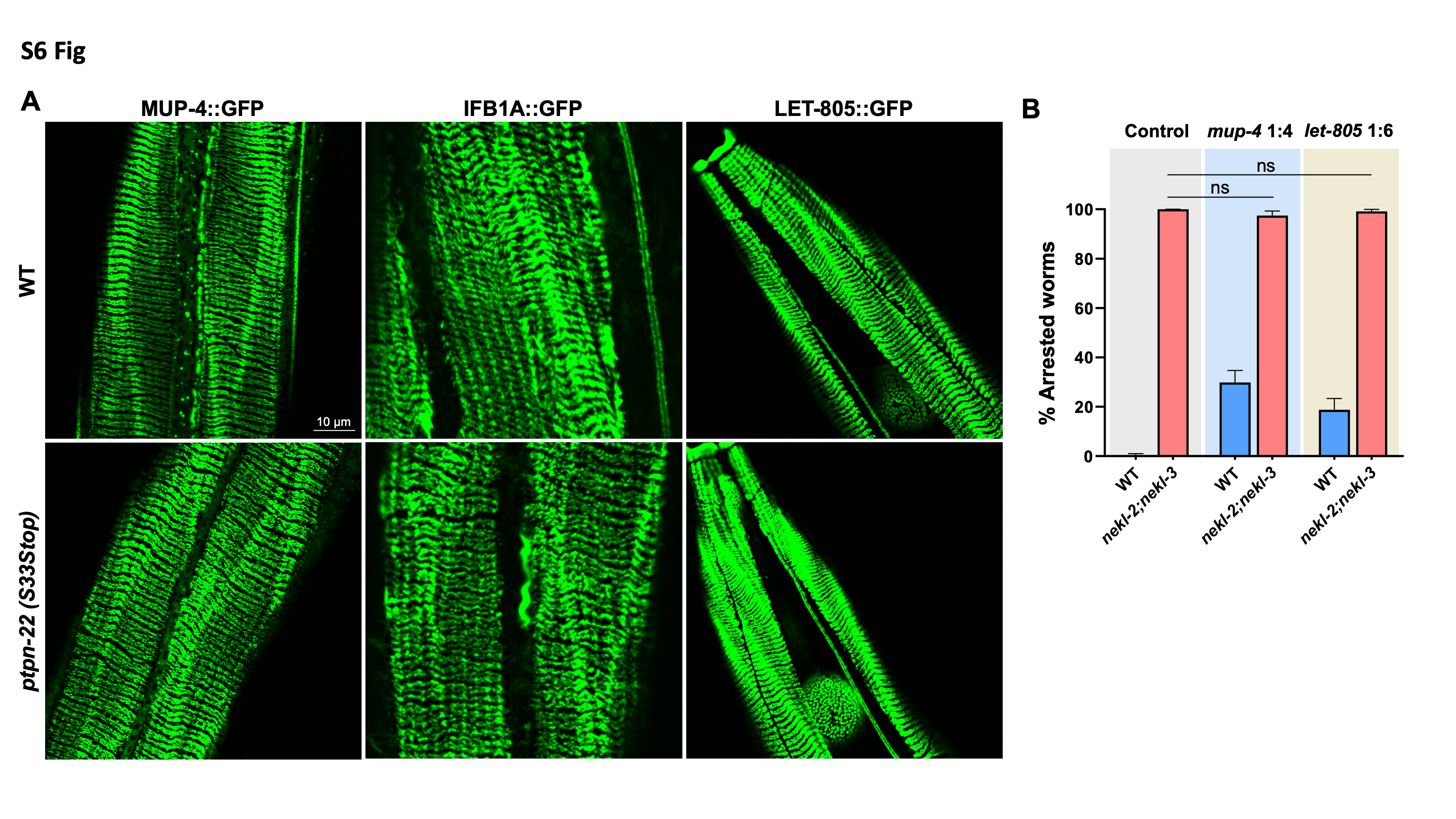

Supplement: S6 Fig — (A) Confocal microscopy images of day-1 adults of the indicated backgrounds expressing MUP-4::GFP, IFB1A::GFP, and LET-805::GFP. Note that no gross differences in the localization of CeHD proteins were detected. (B) RNAi-suppression experiments were carried out with wild-type and nekl-2; nekl-3 mutants after partial knockdown of mup-4 and let-805 using RNAi feeding at different dilutions (see Materials and Methods). Note that no reduction in the percentage of nekl-2; nekl-3 arrest was observed. Error bars represent 95% confidence intervals. Fisher’s exact test was used to calculate p-values; ns, not significant. Raw data are available in the S7 File. (PNG) [file pgen.1011219.s006.png]

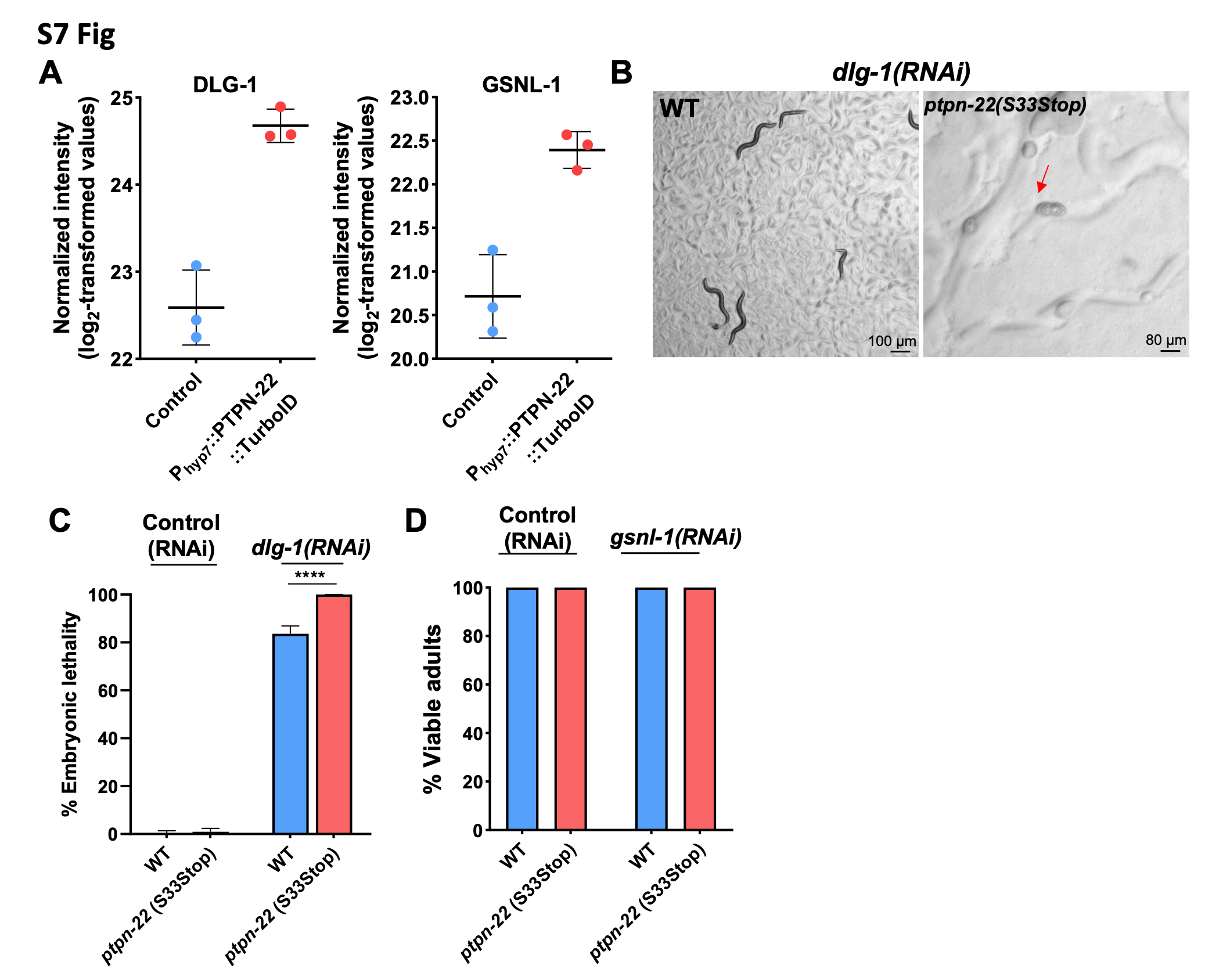

Supplement: S7 Fig — (A) Dot plots show the enrichment of the indicated proteins in the N2 and Phyp7::PTPN-22::TurboID samples. (B) Bright-field images of wild-type and ptpn-22(S33Stop) worms on control (empty vector) or dlg-1 RNAi feeding plates. Red arrow shows the presence of dead eggs on the dlg-1(RNAi) plate. (C, D) Bar graphs show the percentage of embryonic lethality (C) and viable adults (D) of the indicated backgrounds in control (empty vector) and gsnl-1 RNAi feeding plates. Raw data are available in S7 File. (PNG) [file pgen.1011219.s007.png]
