# Supplementary material for: A conserved protein tyrosine phosphatase, PTPN-22, functions in diverse developmental processes in C. elegans
Source: PLoS Genet. 2024 Aug 22;20(8):e1011219. doi: 10.1371/journal.pgen.1011219 (PMC11373843; doi:10.1371/journal.pgen.1011219)
Supplement: S1 File — (PDF) [file pgen.1011219.s008.pdf]

Red = new nucleotide, yellow = mutated nucleotide, * = STOP codon, lowercase letter = intron, capital letter = exon, and green = novel amino acid before the stop codon

ptpn-22(fd269)D239fs

DNA sequence:

GGTGATTACGAGATCACTTTAGAAGAGGAGAAACTCTTTGACATGATGTAATATTTAATGAGATGTCTGATGAATATTTAATGA

Protein sequence:

673 GGTGATTACGAGATCACTTTAGAAGAGGAGAAACTCTTTGAC 714

225 G D Y E I T L E E E K L F D 238

715 ATGATGTAATATTTAATGAGATGTCTGATGAATATTTAATGA 756

239 M M * Y L M R C L M N I * * 252

ptpn-22(fd331)E232fs

sgRNA: TTACGAGATCACTTTAGAAG

Repair template:
AGAACAAGTCGGTGAAAGTGAACTTTTCGGTGATTACGAGATCACTTTAGACTAGTAGAAACTCTTTGACGATGATGAATATTTAATGAGATGTCTAAAAATGGAGA

DNA sequence:

GGTGATTACGAGATCACTTTAAGAAGAGGAGAAACTCTTTGACGATGATGAATATTTAATGAGATGTCTAAAAATGGAGAA

Protein sequence:

673 GGTGATTACGAGATCACTTTAAGAAGAGGAGAAACTCTTTGA 714

225 G D Y E I T L R R G E T L * 238

715 CGATGATGAATATTTAATGAGATGTCTAAAAATGGAGAA 753

239 R * * I F N E M S K N G E 251

dnbp-1(fd385)

sgRNA: ACGTTGCTTCATGTTTTGGA

Repair template:

GGTGGACGATGATGATGTTCCAGGTTTTGAGCCTTTTGGAGACACAGAACCATTAACGTTACTA
CATgttttggatttcctggaagaagaacaagcattaagattaataaaaaatattgtttgcaact

DNA sequence:
GAAATCCAAGCTTTTGGAGTTGCAAACAATATTTTTATTAATCTTAATGCTTGTTCTTCTTCC
AGGAAATCCAAAACATGTAGTAACGTTAATGGTTCTGTGTCT

Protein sequence:

2701 CAGGATCGTATGAAGCCGTTGCAAAAGATTGCGAATAAGGAAATC 2745

901 Q D R M K P L Q K I A N K E I 915

2746 CAAGCTTTTGGAGTTGCAAACAATATTTTTATTAATCTTAATGC 2790

916 Q A F G V A N N I F Y * S * C 930

dnbp-1(fd386)

sgRNA: ACGTTGCTTCATGTTTTGGA

Repair template:

GGTGGACGATGATGATGTTCCAGGTTTTGAGCCTTTTGGAGACACAGAACCATTAACGTTACTA
CATgttttggatttcctggaagaagaacaagcattaagattaataaaaaatattgtttgcaact

DNA sequence:
GCCATCATCATCGTCCACCACTGTCACTGAATCACCTTGGGCAACTGCTCC

Protein sequence:

2701 CAGGATCGTATGAAGCCGTTGCAAAAGATTGCGAATAAGGCCATC 2745

901 Q D R M K P L Q K I A N K A I 915

2746 ATCATCGTCCACCACTGTCACTGAATCACCTTGGGCAACTGCTCC 2790

916 I I V H H C H * I T L G N C S 930

ptpn-22(fd388)S33Stop

sgRNA: TTGACTACTGGATTCGATGT

Repair template:

tgtttttagcctaaattttcgctattttcgaacCATTTGGAGTATCTTTAGACTACTTAAGTC TATGTATTCAGTGGCGGCGGTGGGCTTTTTGGCTTTTGTTCCTGAATTTCctgaaaaa

DNA sequence:

GAAATTCAGGAACAAAAGCCAAAAAGCCCACCGCCGCCACTGAATACATAGACTTAAGTAGTCT AAAGATACTCCAAA

Protein sequence:

43 CCTCCAGAAATTCAGGAACAAAAGCCAAAAAGCCCACCGCCG 84

15 P P E I Q E Q K P K S P P P 28

85 CCACTGAATACATAGACTTAAGTAGTCTAAAGATACTCCAAA 126

29 P L N T * T * V V * R Y S K 42

ptpn-22(fd390)S33Stop

sgRNA: TTGACTACTGGATTCGATGT

Repair template:

tgtttttagcctaaattttcgctattttcgaacCATTTGGAGTATCTTTAGACTACTTAAGTC TATGTATTCAGTGGCGGCGGTGGGCTTTTTGGCTTTTGTTCCTGAATTTCctgaaaaa

DNA sequence:

GAAATTCAGGAACAAAAGCCAAAAAGCCCACCGCCGCCACTGAATACATAGACTTAAGTAGTCT AAAGATATAATTCG

Protein sequence:

43 CCTCCAGAAATTCAGGAACAAAAGCCAAAAAGCCCACCGCCG 84

15 P P E I Q E Q K P K S P P P 28

85 CCACTGAATACATAGACTTAAGTAGTCTAAAGATATAATTCG 126

29 P L N T * T * V V * R Y N S 42

ptpn-22(fd406)C306S

sgRNA: AATCGGTGAAGAATCTTGGG

Repair Template:

GAATAAAATTAATCGCAATAATTGTGCCCGTTCTACCAACACCCGCACTAGAATGTACTAGTAT
CGGTGAAGAATCTTGGGGAGACACTTCTTCATGAATATTCTCCATTAAATCAATCATATTCAAT
AGTTG

DNA sequence:

TGGAGAATATTCATGAAGAAGTGTCTCCCCAAGATTCTTCACCGATACTAGTACATTCTAGTGC
GGGTGTTGGTAGAACGGGCACAATTATTGCGATTAATTTTATT

Protein sequence:

883 TCTCCCCAAGATTCTTCACCGATACTAGTACATTCTAGTGCG 924

295 S P Q D S S P I L V H S S A 308

925 GGTGTTGGTAGAACGGGCACAATTATTGCGATTAATTTTATT 966

309 G V G R T G T I I A I N F I 322

***ptpn-22(fd444)* Proline-rich region mutated using CRISPR**

sgRNA: ATGTAGGCAGTGGCGGCGGT

Repair template:

AgcctaaattttcgctattttcgaacCATTTGGAGTATCTTTGACTACTGCATTCGATGTAGCC
AGTGCCGCAGCTGCGCTTTTTGGCTTTTGTTCCTGAATTTCctgaaaaaaaccaaattttatg

DNA sequence:

GAAATTCAGGAACAAAAGCCAAAAAGCGCAGCTGCGGCACTGGCTACATCGAATGCAGTAGTCA
AAGATACTCCAAATGgttcgaaaatagcgaaaattt

Protein sequence:

43 CCTCCAGAAATTCAGGAACAAAAGCCAAAAAGCGCAGCTGCG 84

15 P P E I Q E Q K P K S A A A 28

85 GCACTGGCTACATCGAATGCAGTAGTC 111

29 A L A T S N A V V 37

ptpn-22(syb6505)

DNA sequence:

CCTATGGGGCCGGAAGATCTTGGCAGCAGCGGCATTTGGAGGTGGTGGATCAGGCTCGGGAG
GTCGAGGCTCAGGATCCGGTTCCGGCTCCGGCTCTGGTTCCGGTTCGGGTTCCGGTTCTGGAAA
GGATAACACCGTTCCACTTAAGCTTATCGCCCTTCTTGCCAACGGAGAATTCCACTCTGGAGAG
CAACTTGGAGAGACTCTTGGAATGTCCCGTGCTGCCATCAACAAGCATATCCAAACCCTTCGTG
ATTGGGGAGTTGATGTTTTCACTGTTCCAGGAAAGgtaagtttaaacatatatatactaactaa
ccctgattatttaaattttcagGGATACTCCCTTCCAGAGCCAATCCCACTTCTTAACGCCAAG
CAAATCCTTGGACAACTTGATGGAGGATCCGTCGCTGTCCTTCCAGTTGTTGATTCCACCAACC
AATACCTTCTTGACCGTATCGGAGAGCTTAAGTCTGGAGACGCCTGCATCGCTGAGTACCAACA
AGCTGGACGCGGATCTCGCGGACGCAAGTGGTTCTCCCCATTCGGAGCCAACCTTTACCTTTCT
ATGTTCTGGCGTCTTAAGCGTGGACCAGCTGCTATCGGACTTGGACCAGTTATCGGAATCGTTA
TGGCTGAGGCCCTTCGTAAGCTTGGAGCTGATAAGgtaagtttaaacagttcggtactaactaa
ccatacatatttaaattttcagGTTCGTGTTAAGTGGCCAAACGATCTTTACCTTCAAGACCGT
AAGCTTGCTGGAATCCTTGTCGAGCTTGCTGGAATCACCGGAGACGCCGCTCAAATCGTTATCG
GAGCTGGAATCAACGTTGCCATGCGTCGTGTTGAGGAGTCTGTTGTTAACCAAGGATGGATCAC
TCTTCAAGAGGCTGGAATCAACCTTGATCGTAACACCCTTGCTGCCACCCTTATCCGTGAGCTT
CGTGCTGCCCTTGAGCTTTTCGAGCAAGAGGGACTTGCCCCATACCTTCCACGCTGGGAGAAGC
TTGACAACTTCATCAACCGCCCAGTTAAGCTTATCATCGGAGATAAGGAAATCTTCGGAATCTC
TCGCGGAATCGACAAGCAAGGAGCTCTTCTTCTTGAGCAAGATGGAGTCATTAAGCCATGGATG
GGAGGAGAGATTTCCCTTCGTTCCGCTGAGAAGGCCGGAGGAGATTATAAAGACGATGACGATA
AGCGTGACTACAAGGACGACGACGACAAGCGTGATTACAAGGATGACGATGACAAGTGAatgtg
agaaggcgctttttagcttgaa
